# T2-weighted MRI defines critical compression in the distal carpal tunnel that is relieved after decompressive surgery

**DOI:** 10.1016/j.bjps.2022.02.039

**Published:** 2022-02-26

**Authors:** S. Tullie, A. Wiberg, D. Furniss, A. Schmid

**Affiliations:** 1 Nuffield Department of Orthopaedics, Rheumatology, and Musculoskeletal Sciences, University of Oxford, Oxford, OX3 7LD, UK; 2 Department of Plastic and Reconstructive Surgery, Oxford University Hospitals NHS Foundation Trust, John Radcliffe Hospital, Oxford, OX3 9DU, UK; 3 Nuffield Department of Clinical Neurosciences, University of Oxford, John Radcliffe Hospital, Oxford, OX3 9DU, UK

**Keywords:** carpal tunnel syndrome, MRI, sub-synovial connective tissue, distal carpal tunnel, wrist index

## Abstract

**Introduction:**

Despite Carpal Tunnel Syndrome (CTS) being the most common entrapment neuropathy, its pathophysiology remains debated. Sub-synovial connective tissues (SSCT) within the carpal tunnel are thought to play a role but are poorly characterised. MRI analysis offers potentially novel insights into SSCT characteristics.

**Methods:**

A pilot study of T2-weighted MRI was performed in healthy controls (n=7), and in CTS patients (n=16) pre- and 6 months post-surgical decompression. Image analysis was performed to quantify SSCT cross-sectional area, SSCT signal intensity ratio, and wrist index (depth/width) at distal, middle and proximal wrist landmarks.

**Results:**

Median SSCT signal intensity was lower in the distal carpal tunnel of CTS patients pre-operatively (0.96) compared to controls (1.13; *P* = 0.008) and normalised post-operatively (1.13, *P* = 0.001). Median wrist index was also lower in CTS patients pre-operatively (0.60) compared to controls (0.67, *P* = 0.022), and again normalised post-operatively (0.74, *P* =0.001). This was attributed to changes in carpal depth in the antero-posterior axis with decompression surgery.

**Conclusion:**

This pilot study successfully demonstrated MRI assessment of SSCT in patients with CTS. The decreased SSCT signal intensities suggest predominant changes at the distal tunnel, potentially indicating reduced SSCT perfusion pre-surgery which normalised post-surgery. Our preliminary findings merit further investigation in a larger cohort.

## Introduction

Carpal Tunnel Syndrome (CTS) is the most common entrapment neuropathy, with an estimated prevalence of 5-10%^1^. CTS symptoms can be very disabling^2^ and patients often require decompressive surgery. The predicted doubling of operations for CTS between 2010 and 2030^3^ poses a significant health economic burden.^4^ It is therefore essential to further understand the complex and multifactorial pathophysiology underlying CTS.

Increased extra-neural pressure is strongly implicated in CTS pathophysiology^5^, with peri-neural structures postulated to impair intraneural circulation, resulting in demyelination and axonal loss^6^. Surgical decompression of the carpal tunnel is successful in the majority of cases^7^; however, a significant sub-group experience further symptoms^7^. The median nerve and the flexor tendons within the carpal tunnel are surrounded by the sub-synovial connective tissues (SSCT). The SSCT is a multilayer and innervated tissue unit composed largely of collagen fibres, blood vessels and lymphatics^8^. It is believed to play a central role in CTS pathophysiology, where forceful or repetitive low velocity movement can cause SSCT microdamage, resulting in thickening and fibrosis^8^. Such fibrotic changes in SSCT architecture are consistent with histological analysis in CTS patients^6,9^, and it is postulated that SSCT fibrosis/thickening causes median nerve tethering^6^, impairing conduction via altered neural microcirculation^10^, structure^11^ and function^12,13^. This SSCT contribution to CTS pathophysiology is supported by overexpression of pro-fibrotic mediators in the SSCT of CTS patients^14^.

In addition to SSCT characteristics, carpal tunnel volume may contribute to CTS pathophysiology, with previous research showing decreased carpal tunnel volumes in CTS^15^, whereas other work identified larger carpal tunnel volumes in CTS patients^16^. These conflicting results led to the suggestion that another anatomical tissue might underlie CTS, such as SSCT^16^. Carpal tunnel morphology as measured by wrist index (carpal tunnel height/width) is one factor found to be significantly increased in CTS patients in ultrasound-based analysis^17^ and in cadaveric studies^18^. Furthermore, post-operative increases in carpal tunnel area following decompression surgery have been linked with symptom resolution^19,20^.

MRI imaging allows for analysis of both SSCT signal characteristics and carpal tunnel morphology, proving valuable in understanding the underlying disease process in CTS. Signal intensity on T2- weighted images are thought to reflect acute inflammation^21^ and tissue blood flow^22^, and images can also be used to quantify wrist index^17,18,23^. In this pilot study, we aimed to identify changes in SSCT and carpal tunnel morphometrics in patients with CTS compared to healthy controls, and determine any changes in these parameters post-operatively in CTS patients. Such changes identified on MRI may contribute to an increased understanding of the underlying pathophysiology in CTS, with SSCT changes in perfusion or area post-operatively of potential clinical significance.

## Methods

### Participants

Patients with clinically and electrodiagnostically confirmed CTS who were listed to undergo decompression surgery were recruited from surgical waiting lists at Oxford University Hospitals NHS Foundation Trust as part of a larger cohort^24^. Patients were excluded if electrodiagnostic testing revealed abnormalities other than CTS; if another medical condition affecting the upper limb/neck was present (e.g. hand osteoarthritis/cervical radiculopathy); if there was a history of trauma to the upper limb and/or neck; if CTS was related to pregnancy or diabetes; or if there was any contraindication to MRI. Patients undergoing repeat carpal tunnel surgery were also excluded. Healthy control participants were recruited using media advertisements and public noticeboard posters within Oxford University Hospital NHS Foundation Trust.

Patients with CTS attended a pre-operative baseline assessment as well as a follow-up appointment 6 months post-operatively for MRI and clinical phenotyping. Healthy volunteers only attended a single session for MRI and clinical phenotyping. We measured the hand to be operated in patients; in healthy controls we measured the non-dominant hand. Ethical approval was granted by the South Central Berkshire B Committee (REC ref 10/H0706/35) and the London Riverside Committee (REC ref 10/H0706/35). All patients gave informed written consent.

### MRI acquisition and analysis

All MRIs were performed on a 3 Tesla whole body MR system (MAGNETOM Prisma, Siemens Healthcare, Erlangen, Germany, 80mT/m) with a 4-channel flexible surface coil (Siemens Healthcare, Erlangen, Germany). Axial gradient echo sequences were acquired using a T2-weighted turbo-spin echo sequence with a 0.4 × 0.4 × 2mm voxel size (TR 9100ms, TE 60ms, FoV 102mm, 180 degrees flip angle, bandwidth 181Hz/Px, echo spacing 12ms, turbo factor 16, scan time 5 minutes). Participants were positioned in the ‘Superman position’.

**Figure 1:**
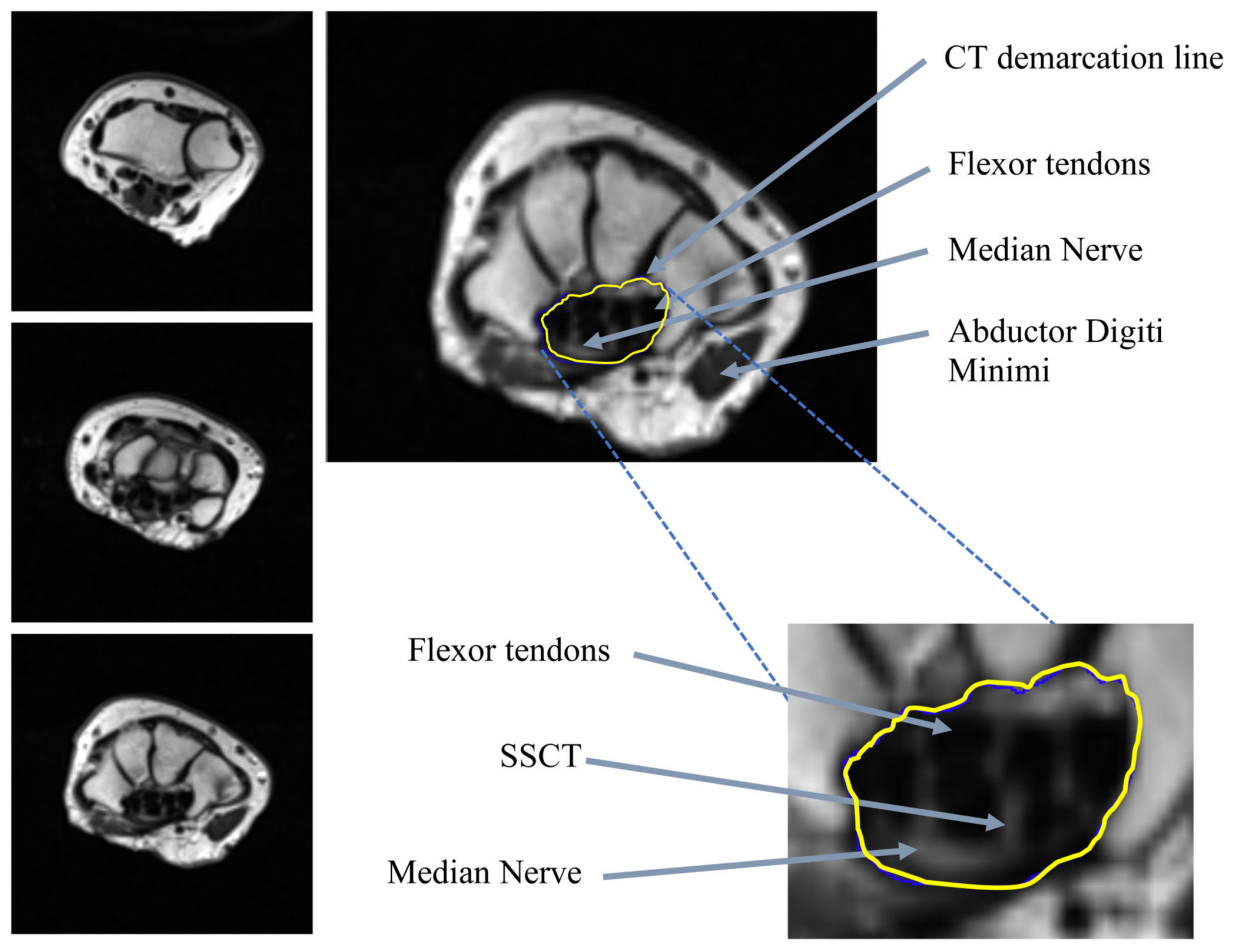
***Left panel:*** T2-weighted MRI at the proximal wrist landmark (top image), middle landmark (middle image), distal landmark (bottom image). ***Right panel:*** Top: T2-weighted MRI with the carpal tunnel demarcated at distal landmark (yellow line). Labels demonstrating median nerve, flexor tendons, and abductor digiti minimi (used for intensity normalization). *Bottom:* ‘zoomed-in’ image showing SSCT visible as tissue surrounding flexor tendons and median nerve within the demarcated carpal tunnel region.

T2-weighted image analysis was performed using Fiji (ImageJ, version 1.52p)^25^ to focus on signal intensities and cross-sectional areas of the SSCT (Figure 1). Analysis was performed by a single assessor (ST) at three pre-defined anatomical landmarks (proximally: distal radio-ulnar joint, mid-carpal tunnel: pisiform bone, distal carpal tunnel: hook of hamate). At each landmark, three axial slices were analysed and averages used for analyses. The assessor was blinded to case/control and pre-/post-operative status.

Individual regions-of-interest (ROIs) were used to manually demarcate the median nerve and each flexor tendon. A single further ROI was used to identify the entire carpal tunnel. The nerve and tendon cross-sectional areas were then subtracted from the cross-sectional area of the carpal tunnel. The resulting value gave the cross-sectional area of the SSCT within the carpal tunnel at each landmark.

Mean signal intensities were calculated for the SSCT area by dividing the sum of per-pixel signal intensity of the SSCT by the area of the SSCT. As signal depends on coil position, signal intensities were normalised to non-median nerve innervated muscle (proximal landmark: pronator quadratus, middle/distal landmarks: abductor digiti minimi) to create a signal intensity ratio. Wrist index^17^ was measured at the two landmarks within the carpal tunnel at the levels of the pisiform (middle) and the hook of hamate (distal) using carpal depth and width values measured perpendicularly at the point of greatest carpal depth.

Reliability analysis was performed across all data collected with the image extraction technique using an intraclass correlation coefficient (ICC) generated from a two-way mixed-effect model (based on multiple ratings and absolute agreement). This assessed reliability of the data extractor on a previously established scale^26^ by assessing the correlation and repeatability of triplicate values that were extracted for each parameter measured at each carpal tunnel landmark.

### Statistical analysis

Data were analysed using SPSS Statistics (Version 27.0.1.0, IBM) and GraphPad Prism (version 9.0.2). Imaging and clinical phenotype data were non-parametric, so we used Mann-Whitney U tests to compare non-paired data (patients versus healthy controls) and Wilcoxon tests for paired data (pre- and post-CTS surgery). P-values < 0.05 were judged to be statistically significant in this exploratory analysis. As this was a pilot study, no power calculations were performed, but our results will help inform future work.

## Results

The study cohort included 16 patients with CTS and 7 healthy controls with their characteristics summarised in Table 1. ICCs of MRI measurements demonstrated good to excellent reliability with full ICCs summarised in Supplemental Table 1.

### SSCT signal intensity ratio

**Figure 2:**
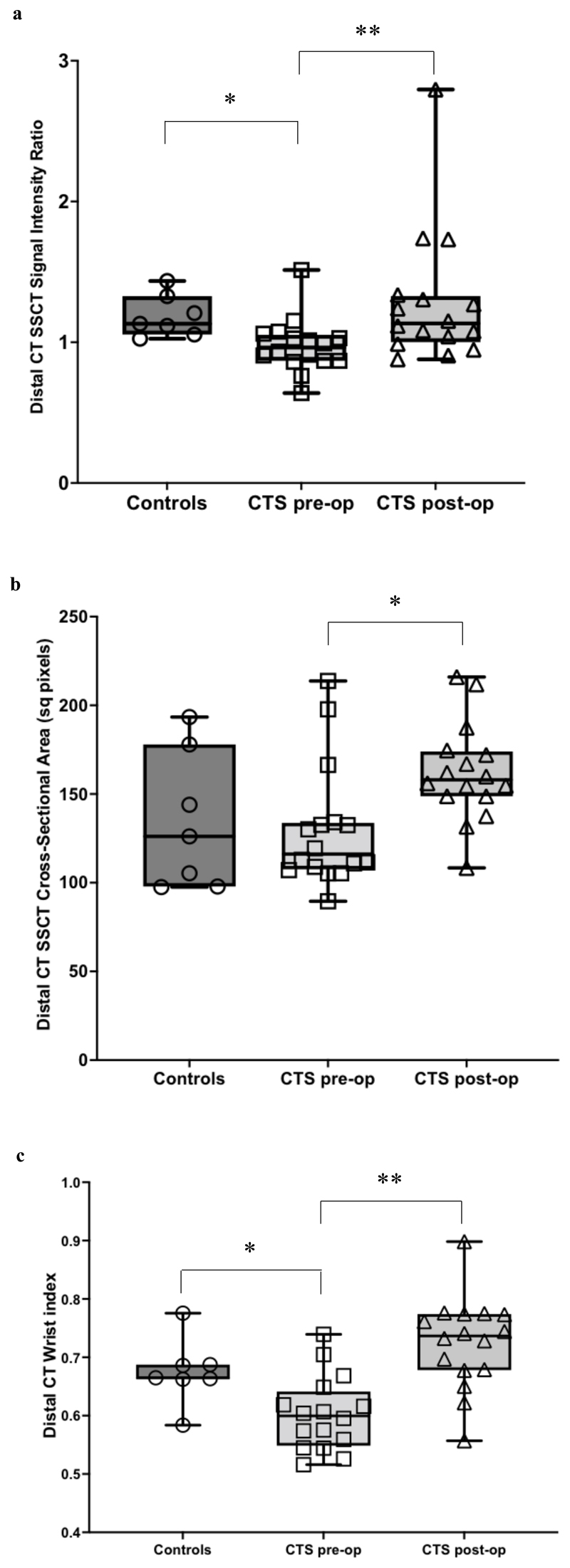
a: SSCT signal intensity ratio in the distal carpal tunnel of healthy controls, CTS patients pre-operatively and CTS patients post-operatively. *p=0.008, controls v CTS pre-op. **p=0.001 CTS pre-op v CTS post-op. b: SSCT cross-sectional area in the distal carpal tunnel of healthy controls, CTS patients pre-operatively and CTS patients post-operatively. *p=0.006, CTS pre-op v CTS post-op. c. Comparison of wrist index in the distal carpal tunnel of healthy controls, CTS patients pre-operatively and CTS patients post-operatively. *p=0.022, controls v CTS pre-op. **p=0.001, CTS pre-op vs CTS post-op. Box plots represent median values and interquartile range, whiskers represent full data range, and all individual data points are shown

Median SSCT signal intensity ratio was lower in the distal carpal tunnel of CTS patients pre-operatively compared to healthy controls, but not at the middle carpal tunnel or proximal landmarks (Table 2, Figure 2a). This decreased signal intensity ratio normalised after carpal tunnel release (Table 3, Figure 2a).

### SSCT cross-sectional area

Paired comparison of CTS patients pre- and post-operatively found a significant increase in SSCT cross-sectional area at the distal carpal tunnel landmark (Table 3, Figure 2b). No significant changes were identified in the SSCT cross-sectional areas of CTS patients at the proximal or middle carpal tunnel landmarks (Table 3). Similarly, we found no differences between SSCT area between pre-operative CTS patients and healthy controls (Table 2).

### Wrist Index

In CTS patients pre-operatively, median wrist index at the distal carpal tunnel landmark was lower than that of healthy control subjects (Table 2, Figure 2c), and normalised following carpal tunnel release (Table 3, Figure 2c). Median wrist index at the middle carpal tunnel landmark also increased post-operatively in CTS patients (Table 2). At both landmarks the dominant influence underlying post-operative wrist index change was carpal depth increase, with the mean carpal depth percentage increase greater than the mean carpal width percentage decrease at the middle [mean carpal depth increase (SD): 8.06% (8.07%), mean carpal width decrease (SD): 0.76% (8.30%)] and distal carpal tunnel [mean carpal depth increase (SD): 15.4% (9.85%), mean carpal width decrease (SD): 3.83% (9.74%)]. Further analysis of carpal width and depth changes on an individual level reinforced this finding, with 12/15 CTS patients demonstrating a post-operative increase in wrist index at the distal carpal tunnel landmark found to be the result of a proportionally greater carpal depth increase compared to carpal width decrease. All of the 12 CTS patients demonstrating post-operative increases in wrist index at the middle carpal tunnel landmark were found to have a proportionally greater carpal depth increase compared to carpal width decrease.

## Discussion

Despite significant research into CTS pathophysiology, relatively little is understood about the contribution of SSCT to the disease,^8,27^ with no previous studies analysing SSCT pre- and post-operatively using MRI. Our findings indicate the distal carpal tunnel seems to be particularly constrictive in CTS, with decreased signal intensity likely representing reduced SSCT perfusion, which returns to normal post-operatively. This correlates with an increase in wrist depth, and therefore wrist index, demonstrating decreased tissue compression in the antero-posterior dimension. This emphasises the importance of successfully decompressing the distal carpal tunnel to avoid persistent CTS symptoms^28^, and the data collected merits further investigation to assess the role of SSCT in CTS pathophysiology.

Even in this relatively small pilot cohort, SSCT intensity changes are significant on MRI, representing a potentially important region for further exploration. SSCT signal intensity ratio was lower in the distal carpal tunnel of CTS patients pre-operatively compared to healthy control subjects. Therefore, there does not appear to be greater acute inflammation in the SSCT of CTS patients akin to elevated T2 median nerve signal reflecting nerve inflammation^29^. Instead, lower T2 MRI signal intensity may demonstrate decreased perfusion in the tightly compressed distal carpal tunnel of CTS patients, consistent with previous contrast enhanced MRI analysis of median nerve^22^. The return of distal carpal tunnel signal intensity ratios to control levels six months post-operatively suggests restoration of the SSCT circulation to its normal physiological state.

Alternatively, these findings may provide insight into the type of inflammation present in the SSCT of CTS patients. Lowered signal intensity ratio is more characteristic of chronic tissue inflammation^30^, a finding that would be expected in our CTS cohort with longstanding diagnoses of neuropathy. Such changes could include fibrosis, a characteristic that is hypo-intense on T2-weighted MRI in carpal tunnel scarring^30^, widely reported in previous SSCT histology^6^, and consistent with previously proposed models of SSCT in CTS^8,27^. This fibrotic extra-neural environment causes median nerve tethering and altered tissue microcirculation, contributing to the pathophysiological changes underlying CTS^11–13^. Tissue remodelling post-operatively in the SSCT could reduce fibrosis, relieve median nerve tethering, and allow for restoration of normal extra-neural tissue blood flow^31^.

Previous conflicting research into whether carpal tunnel area *per se* had a role in CTS led to the suggestion that the cross-sectional area volume of the SSCT contained within the carpal tunnel might be the main anatomical influence on CTS pathophysiology^15,16,32^. However, no significant difference in SSCT cross-sectional area was demonstrated between CTS patients and controls. SSCT cross-sectional area was significantly increased in the distal carpal tunnel of CTS patients post-operatively, possibly reflecting decreased confinement post-decompression at a previously described focal point of median nerve impingement^32–34^.

Our analysis of wrist index found changes in the carpal tunnel at the middle and distal landmarks that might provide insight into tissue morphology. Wrist index was lower in CTS patients pre-operatively relative to healthy controls at the distal carpal tunnel, a finding at odds with previous research, where ultrasound identified higher wrist index values in CTS patients^17^. These conflicting findings could be the result of our study being underpowered; alternatively, they could indicate that MRI is a more accurate tool for wrist index measurement, as our use of cross-sectional imaging excluded the wrist soft tissues that were included in the ultrasound-based measurements. In support of this, our CTS patients demonstrated significant increases in wrist index post-operatively, with values returning to those equivalent to healthy controls. Individual analysis of these changes found carpal depth increase to be the major influential factor in post-operative wrist index increases in CTS patients. This is consistent with post-operative wrist index changes in CTS patients in previous MRI-based research^20^ where increased carpal depth underlying wrist index increase was postulated to be a protective mechanism for the median nerve. Wrist index was also found to increase in the carpal tunnel at the middle landmark, although this increase was less marked than at the distal carpal tunnel. This suggests surgical decompression allows for greater antero-posterior carpal tunnel expansion, which, in turn, relieves median nerve impingement^18,20^, specifically in the distal carpal tunnel.

The primary strength of this research is its original exploration of SSCT using MRI, with pre- and post-operative SSCT imaging changes not having been described previously. Previous imaging analysis of SSCT has been limited to ultrasound, with analysis focused on tissue dynamics rather than more detailed tissue characterisation^9^. Although carpal tunnel ultrasound is clinically useful, particularly when performed by an experienced hand surgeon or MSK Radiologist, it remains a highly operator-dependent modality^35^. MRI offers less operator dependency and has been used to investigate CTS, but predominantly in characterising the pathological changes in the median nerve alone^36,37,38^. This study adds to the literature detailing pre- and post-operative wrist index findings in patients^20^ with previous findings from cadaveric studies^18,39^. Limitations are rooted in sample size, limiting the power to detect statistically significant differences. This study represents a secondary analysis of an existing cohort so scan availability was limited. Considering the sample size available, this study represents a successful exploratory investigation into the use of MRI in identifying changes in SSCT that are of relevance to CTS. Future studies need to include additional MRI sequences to further detangle inflammatory from ischaemic mechanisms.

## Conclusion

Using T2-weighted MRI in patients with carpal tunnel syndrome pre- and post-operatively and comparing these to healthy controls, we have demonstrated significant differences in the SSCT and wrist index at the distal carpal tunnel. This emphasises the importance of adequate decompression of this section of the carpal tunnel, consistent with previous clinical studies. This pilot work opens up opportunities for further research into the specific role of the SSCT in CTS, including further imaging and molecular studies. In particular, it will be interesting to evaluate patients with persistent or recurrent symptoms.

## Supplementary Material

Supplementary Table 1

## Figures and Tables

**Table 1:** Demographic and clinical data of healthy control subjects and patients with carpal tunnel syndrome (CTS).

	CTS	Healthy controls
**Number of participants**	16	7
**Gender, female/male**	14/2	4/3
**Age, years (mean, SD)**	59.3 (10.4)	61.6 (11.1)
**BMI, kg/m^2^ (mean, SD)**	26.2 (4.84)	25.0 (3.17)
**Symptom duration, months (mean, SD)**	50.6 (39.1)	n/a
**Electrodiagnostic testing grade (median, IQR)**	3 (1)	0
**Boston scale (mean, SD)**
**Symptoms**	2.61 (0.61)	1 (0)
**Function**	2.27 (0.86)	1 (0)

**Table 2:** Comparison of median SSCT cross-sectional area, signal intensity ratio, and wrist index between healthy controls and CTS patients, at three carpal tunnel landmarks.

	Proximal		Middle			Distal		
	*Area (sq pixels)*	*Signal Intensity Ratio*	*Area (sq pixels)*	*Signal Intensity Ratio*	*Wrist Index*	*Area (sq pixels)*	*Signal Intensity Ratio*	*Wrist Index*
**Controls**	197.0 (45.4)	1.66 (0.46)	158.9 (18.4)	0.98 (0.50)	0.67 (0.11)	126.1 (59.4)	1.13 (0.18)	0.67 (0.02)
**CTS pre-op**	228.9 (76.4)	1.95 (0.40)	197.7 (72.5)	1.00 (0.46)	0.69 (0.05)	116.1 (24.5)	0.96 (0.17)	0.60 (0.07)
**p-value**	0.154	0.278	0.118	0.579	1.000	1.000	***0.008***	***0.022***

Figures expressed as median (IQR). Significant p-values are shown in ***bold italics***.

**Table 3:** Comparison of median SSCT cross-sectional area, signal intensity ratio, and wrist index between CTS patients pre- and post-operatively, at three carpal tunnel landmarks.

	Proximal		Middle			Distal		
	*Area (sq pixels)*	*Signal Intensity Ratio*	*Area (sq pixels)*	*Signal Intensity Ratio*	*Wrist Index*	*Area (sq pixels)*	*Signal Intensity Ratio*	*Wrist Index*
**CTS pre-op**	228.9 (76.4)	1.95 (0.40)	197.7 (72.5)	1.00 (0.46)	0.69 (0.05)	116.1 (24.5)	0.96 (0.17)	0.60 (0.07)
**CTS post-op**	250.3 (44.1)	2.09 (0.59)	204.2 (37.2)	1.14 (0.18)	0.74 (0.08)	158.0 (23.9)	1.13 (0.28)	0.74 (0.09)
**p-value**	0.756	0.379	0.836	0.179	***0.013***	***0.006***	***0.001***	***0.001***

Figures expressed as median (IQR). Significant p-values are shown in ***bold italics***.
