# Supplementary material for: T2-weighted MRI defines critical compression in the distal carpal tunnel that is relieved after decompressive surgery
Source: J Plast Reconstr Aesthet Surg. Author manuscript; Available in PMC 2022 Jul 8. (PMC7613040; doi:10.1016/j.bjps.2022.02.039)
Supplement: Supplementary Table 1 [file EMS143797-supplement-Supplementary_Table_1.pdf]

Supplementary Table 1. Summary of reliability analysis measured using an intraclass correlation coefficient (ICC).

	Number	ICC (95% CI)
**Proximal CT Area**	23	0.95 (0.90-0.98)
**Middle CT Area**	23	0.93 (0.87-0.97)
**Distal CT Area**	23	0.89 (0.79-0.95)
**Proximal CT SI**	23	0.97 (0.94-0.99)
**Middle CT SI**	23	0.90 (0.81-0.96)
**Distal CT SI**	23	0.87 (0.71-0.94)
**Middle CT Carpal Depth**	23	0.95 (0.90-0.98)
**Middle CT Carpal Width**	23	0.97 (0.95-0.99)
**Distal CT Carpal Depth**	23	0.91 (0.83-0.96)
**Distal CT Carpal Width**	23	0.94 (0.88-0.97)
**ADM SI**	23	0.98 (0.97-0.99)
**PQ SI**	22	0.85 (0.72-0.93)
**Post-op Proximal CT Area**	16	0.98 (0.95-0.99)
**Post-op Middle CT Area**	16	0.88 (0.75-0.95)
**Post-op Distal CT Area**	16	0.93 (0.84-0.97)
**Post-op Proximal CT SI**	16	0.98 (0.95-0.99)
**Post-op Middle CT SI**	16	0.95 (0.89-0.98)
**Post-op Distal CT SI**	16	0.98 (0.96-0.99)
**Post-op Middle CT Carpal Depth**	16	0.98 (0.95-0.99)
**Post-op Middle CT Carpal Width**	16	0.98 (0.96-0.99)
**Post-op Distal CT Carpal Depth**	16	0.95 (0.90-0.98)
**Post-op Distal CT Carpal Width**	16	0.97 (0.94-0.99)
**Post-op ADM SI**	16	0.98 (0.96-0.99)
**Post-op PQ SI**	16	0.95 (0.88-0.98)

Intraclass correlation coefficient (ICC) performed, with 95% confidence intervals (95% CI) expressed, at proximal, middle and distal carpal tunnel (CT) landmarks for all parameters measured. Parameters measured included area, signal intensity (SI), carpal width, carpal depth, abductor digiti minimi signal intensity (ADM SI), and pronator quadratus signal intensity (PQ SI). These were repeated for post-operative CTS patient data measures (post-op).
